# Experience Within the Emergency Department and Improved Productivity for First-Year Residents in Emergency Medicine and Other Specialties

**DOI:** 10.5811/westjem.2017.10.34819

**Published:** 2017-12-14

**Authors:** Joshua W. Joseph, David T. Chiu, Matthew L. Wong, Carlo L. Rosen, Larry A. Nathanson, Leon D. Sanchez

**Affiliations:** * Beth Israel Deaconess Medical Center, Department of Emergency Medicine, Boston, Massachusetts; † Harvard Medical School, Boston, Massachusetts

## Abstract

**Introduction:**

Resident productivity is an important educational and operational measure in emergency medicine (EM). The ability to continue effectively seeing new patients throughout a shift is fundamental to an emergency physician’s development, and residents are integral to the workforce of many academic emergency departments (ED). Our previous work has demonstrated that residents make gains in productivity over the course of intern year; however, it is unclear whether this is from experience as a physician in general on all rotations, or specific to experience in the ED.

**Methods:**

This was a retrospective cohort study, conducted in an urban academic hospital ED, with a three-year EM training program in which first-year residents see new patients ad libitum. We evaluated resident shifts for the total number of new patients seen. We constructed a generalized estimating equation to predict productivity, defined as the number of new patients seen per shift, as a function of the week of the academic year, the number of weeks spent in the ED, and their interaction. Off-service residents’ productivity in the ED was analyzed in a secondary analysis.

**Results:**

We evaluated 7,779 EM intern shifts from 7/1/2010 to 7/1/2016. Interns started at 7.16 (95% confidence interval [CI] [6.87 – 7.45]) patients per nine-hour shift, with an increase of 0.20 (95% CI [0.17 – 0.24]) patients per shift for each week in the ED, over 22 weeks, leading to 11.5 (95% CI [10.6 – 12.7]) patients per shift at the end of their training in the ED. The effects of the week of the academic year and its interaction with weeks in the ED were not significant. We evaluated 2,328 off-service intern shifts, in which off-service residents saw 5.43 (95% CI [5.02 – 5.84]) patients per nine-hour shift initially, with 0.46 additional patients per week in the ED (95% CI [0.25 – 0.68]). The weeks of the academic year were not significant.

**Conclusion:**

Intern productivity in EM correlates with time spent training in the ED, and not with experience on other rotations. Accordingly, an EM intern’s productivity should be evaluated relative to their aggregate time in the ED, rather than the time in the academic year.

## INTRODUCTION

Resident productivity in emergency medicine (EM) is an important educational and operational measure. The ability to continue seeing new patients throughout a shift is fundamental to an emergency physician’s development, reflected in the multitasking (task-switching) milestone of EM training, and can be used as a means of evaluating an individual resident’s progress and competency.^1^ Specifically, the milestone charts a resident’s progress from managing a single patient amid distractions, to being able to manage multiple patients, and eventually to managing the patient volume of the emergency department (ED) itself.^1^ Having a robust and quantifiable means of measuring progress along this milestone would allow educators to identify residents who would benefit from early, targeted interventions to hone their strategy for managing patients and workflow. Operationally, understanding a resident’s capability to see patients is essential to determine appropriate staffing ratios so that residents can have adequate opportunities in which to build their clinical skills and provide the safest and most efficient care possible to patients.

Our previous work has demonstrated that EM residents make steady gains in productivity over the course of their first (intern) year,^2^ while the gains seen between subsequent years of residency are smaller and not evenly distributed over time, which has been well-established in prior studies of resident productivity.^3^–^7^ It remains unclear whether the steady increase in productivity over intern year is a general effect of training as a physician, or specific to experience in the ED. While there are substantial differences in the content and structure of EM training programs relative to those in other fields such as internal medicine or obstetrics, many of the tasks expected of interns, such as performing and communicating a reliable history and physical, are core clinical skills common to many specialties, which trainees may generally perform with greater efficiency and confidence over time, regardless of the specific clinical setting in which they are practicing. Similarly, many non-clinical skills that might affect a physician’s efficiency, such as the ability to navigate a hospital’s electronic health record and computerized order-entry system, may generally improve when working across different areas of a hospital.

We sought to determine whether EM residents’ gains in productivity over the course of their intern year correlated with the aggregate time they had spent in the ED, with the amount of time that had progressed in the academic year, or if there was a more complex relationship between the two. As off-service (non-EM) residents spend a much smaller amount of time in the ED, but an equivalent amount of time in overall training, we sought to evaluate their productivity in a secondary analysis. If gains in productivity are largely determined by a resident’s overall clinical experience, off-service residents should demonstrate similar gains in productivity to EM interns over the course of the academic year.

**Population Health Research Capsule**

What do we already know about this issue?

Productivity, as measured by the number of patients a resident sees over the course of an emergency department shift, tends to improve over the course of training.

What was the research question?

Are improvements in productivity a result of time training in the ED, or on other rotations?

What was the major finding of the study?

Improvements in resident productivity in the ED are only associated with training there.

How does this improve population health?

Understanding rotation schedules’ effect on resident productivity can make schedules more equitable and ensure timely care for patients throughout the academic year.

## METHODS

This was a retrospective cohort study, conducted in an urban academic hospital ED with a three-year EM training program and approximately 55,000 visits per year. In our ED, interns assign themselves to new patients ad libitum. We evaluated consecutive resident shifts for the total number of new patients seen. Resident shifts at our institution are typically nine hours in length, with the last hour primarily to facilitate physician signout. At the beginning of the study period, a portion of interns also participated in longer, 11-hour shifts, which were constrained to a low-acuity area of the ED. These shifts were conducted in an area of the ED with substantially lower nurse and tech staffing, which severely limited residents’ productivity; thus, we did no include these shifts in the final analysis. Off-service interns include those in internal medicine, obstetrics, neurology, anesthesia, and surgery transition years. EM interns at our program rotate in our hospital’s ED for 22 weeks, while off-service interns generally rotate for two weeks, with some surgical and obstetrics interns rotating for three.

We abstracted resident productivity data from a central database of patient assignment timestamps from July 1, 2010, to July 1, 2016. Patient identifiers were not included, and specific patient timestamps (a form of protected health information) were abstracted into aggregate physician-shift data to meet HIPAA Safe Harbor criteria.^8^ We anonymized individual residents’ identities. Physician-shifts were verified with a set of algorithms that examined for logical outliers (e.g., assignments to off-shift residents). We tracked changes in the physician assigned to a patient by our ED dashboard system, which corrects for multiple residents attempting to sign up for the same patient simultaneously or in succession, ensuring that the resident who performs the ultimate evaluation of the patient is the resident of record. The study was exempted by our institution’s review board.

We measured our primary outcome measure of resident productivity in terms of the total number of new (non-signout) patients a resident saw per shift, recorded by the timestamps of when the resident signed up as the resident of record for a patient on our ED information system. Both the number of patients seen and relative value units (RVUs) have been used as measures of productivity in prior studies of resident and attending productivity in EM, reported alternatively as a total over a shift or as an hourly average. Each measure has relative strengths and weaknesses – patients seen directly reflects patient volumes but does not reflect patient complexity, whereas RVUs can reflect patient complexity and the time and effort involved in procedures, but can vary substantially with a physician’s documentation and its interpretation by the individual coding it. Our study examines the total number of patients seen rather than RVUs due to the fact that the RVUs tied to a number of common ED procedures (such as laceration repairs) changed substantially between years of the study period.^9^

We performed statistical analysis with Python 3.5, using the SciPy ecosystem of packages for scientific computing and statistical analysis.^10^–^13^

We assessed our primary outcome measure, the total number of patients seen per resident shift, via a multivariate Gaussian regression using a generalized estimating equation (GEE). While sharing many features of traditional multivariate regression, GEEs are particularly well suited to datasets in which multiple longitudinal measurements are taken of the same subject, which in our case were groups of consecutive shifts by the same resident physician. In particular, GEEs give robust estimations of population-wide effects, even if the time-dependence between repeated observations is unknown or incorrectly specified.^14^ We conservatively defined the covariance matrix for the model as exchangeable. The variables, based on their clinical and educational significance, were the week of the academic year and the number of weeks spent in the ED. As these variables are related, we also included their interaction term as an explicit variable. The interaction term reflects whether there is a change in the association with the number of weeks a resident has spent in the ED over the course of the year (i.e., whether a week in the ED in October is the same as a week in the ED in April, after a resident has completed many more off-service rotations). A two-tailed p- value < 0.05 was considered statistically significant.

## RESULTS

We evaluated 7,779 first-year EM resident shifts from 7/1/2010 to 7/1/2016. Characteristics of the shifts are detailed in Table 1. First-year EM residents (Table 2) saw 7.16 (95% confidence interval [CI] [6.87 – 7.45]) patients per shift at the beginning of their training, with an increase of 0.20 (95% CI 0.17 – 0.24]) patients per shift with each week in the ED, leading to 11.5 (95% CI [10.6 – 12.7]) patients per shift at the end of their 22 weeks of training in the ED. The week of the academic year was not associated with any improvement in productivity, and the interaction between weeks in the ED and the week of the academic year was not significant.

Our secondary analysis evaluated 2,328 shifts of off-service interns working in the ED (Table 3). These interns saw on average 5.43 (95% CI [5.02 – 5.84]) patients per shift when starting in the ED, with 0.46 (95% CI [0.25 – 0.68]) additional patients per shift for each successive week of training in the ED, leading to a total of 6.35 (95% CI [5.52 – 7.82]) at the end of a typical rotation. The week of the academic year again was not significant, nor was the interaction with weeks in the ED.

## DISCUSSION

The development of EM resident productivity over the course of training has been examined by a number of studies, which have consistently found that the greatest increases in productivity occur during intern year, regardless of the measure used to evaluate productivity (such as patients per hour or RVUs).^2^–^7^,^15^ When viewed in terms of patients per hour (the most common resident productivity metric in the literature) our findings of 0.90 (95% CI [0.86 – 0.93]) patients per hour at the beginning of the year are similar to those seen for EM interns in prior studies, which have ranged from as low as 0.73 (95% CI [0.62 – 0.94])^3^ to as high as 1.11 (95% CI [1.02 – 1.20]).^16^

The transition from slow, steady improvements across intern year, to much less consistent improvements across more senior years of residency seen in prior studies may reflect the fact that novices within a field can make relatively rapid gains as they progress from the step-by-step performance of fundamental tasks, to performing them relatively automatically.^17^ The gains made by more experienced trainees in terms of efficiency are smaller, and potentially focused elsewhere, such as in managing more difficult cases, or providing support and teaching to more junior colleagues.

Progression of resident clinical responsibilities may provide another potential explanation for the diminishing productivity gains after intern year seen in prior studies. Within our institution’s EM residency program, patients with unstable vital signs are preferentially assigned to more senior residents immediately after they arrive at triage.^18^ The addition of many more acute patients to senior residents’ workloads, who are more likely to require procedures, consultations, and re-evaluations, increases the complexity residents face with each patient workup. Similarly, the irregular intervals between when these patients arrive increases the number of potential interruptions that residents face, and may complicate strategies that allow interns to increase their productivity, such as trying to see several patients in close geographic proximity to one another. In light of these constraints, it would be difficult for residents starting their second year to continue making linear gains in productivity.

The substantial difference in initial productivity between EM residents and off-service residents may reflect the fact that even basic tasks, such as conducting a history and physical, may vary substantially between EM and other fields. The EM milestones specifically delineate that a more advanced history and physical examination is a focused one.^1^ In comparison, the history and physical typically practiced by an internal medicine resident for an admission may be much more comprehensive than is needed in an ED evaluation.

While many specialties require trainees to carry out focused evaluations in certain situations, such as in closely-scheduled clinic appointments, these evaluations may be structured differently than those conducted during ED evaluations. For instance, the types of problems an internal medicine resident typically sees when caring for patients in clinic may center more on preventative care or management of existing conditions, with fewer evaluations of undifferentiated acute complaints, such as chest pain. Similarly, the evaluations performed in clinic are generally sequential, rather than concurrent, as is often required for managing ED patients, which likely require different skills and heuristics.^6^,^9^,^20^

One potential explanation for EM residents’ initial advantage in productivity is simply that they have had previous experience with EM by virtue of their medical student rotations. Medical students who successfully match in EM are often advised to have two rotations in EM prior to residency,^21^–^23^ which typically entail 14 shifts each.^24^ Accordingly, our model suggests that after a month of experience in the ED (typically 27–28 shifts), off-service residents will perform at an equivalent level to that at which their EM resident peers start.

Operationally, our study suggests that an EM intern who has his/her first ED shifts in November is functionally the equivalent of an EM intern starting in the ED in July. Program directors should plan accordingly to ensure that residents have balanced rotation schedules; otherwise, the program’s ED will face substantial gaps in throughput later in the year. Similarly, the semi-annual nature of Clinical Competency Committee resident reviews may risk unfairly evaluating residents with late ED rotations relative to their peers. If resident schedules cannot be evenly distributed due to scheduling constraints, program directors may consider staggering resident reviews to evaluate their residents at a point when they have had equivalent amounts of time in the ED. EM faculty should be made aware of the potential experience gap when evaluating interns at different times during the academic year.

While our findings show a strong correlation between time spent in the ED and interns’ productivity, and do not show evidence of a ceiling effect to the association at 22 weeks, this does not suggest that residents need additional time in the ED during their intern year. Rotations outside of the ED fulfill important roles within EM training, which aren’t necessarily reflected in terms of productivity. Although two interns who both have spent two months in the ED may demonstrate similar productivity, if one of them has already had rotations in intensive care and anesthesia, his comfort in dealing with critically ill patients and performing procedures may be very different.

## LIMITATIONS

Our study was conducted at a single academic ED at an urban tertiary care center. The ad libitum structure of intern patient assignments at our site may not reflect productivity at programs in which residents are assigned patients by a supervising physician or on a rotational basis, and may therefore underestimate resident physicians’ capacity based on their willingness to see additional patients. Our model does not specifically address the burdens of patient signout or the quantity of patient arrivals per shift; however, intern schedules are designed to maximize the fairness of shift distributions; thus, interns’ shifts will reflect a relatively balanced distribution of busy shifts. Our model also does not address the potential effects of shift length on productivity, as longer shifts have been associated with diminishing productivity.^25^

Given the substantial association between experience in the ED and productivity demonstrated in our study, our model does not take into account the volume of time spent in EM rotations prior to starting residency, which may affect a resident’s baseline productivity. Finally, as all of our EM interns rotate in our ED for 22 weeks, we cannot assess whether there is a potential ceiling effect for additional weeks of ED training, apart from that imposed by graduating to the second-year role. We welcome further research on this topic from programs where interns have more time within their primary ED setting to see if the effect continues.

## CONCLUSION

EM interns’ productivity in the ED correlates with the they have spent training in the ED, and does not appear to be affected by time spent on off-service rotations. Accordingly, their productivity should be evaluated relative to the aggregate time they have in the ED, rather than the time in the academic year. While we believe that productivity is an important measure of a resident’s clinical progression, we encourage further research to help establish measurable outcomes of the other milestones, and to identify strategies that can help residents improve their productivity throughout training.

## Figures and Tables

**Table 1:** Characteristics of residents and shifts evaluated.

Characteristic	N (%)
Residents	441
Emergency Medicine (22 weeks in ED)	77 (17.5%)
Off-Service	364 (82.5%)
Medicine (2 weeks in ED)	286 (78.6%)
Obstetrics (3 weeks in ED)	27 (7.4%)
Podiatry (3–4 weeks in ED)	10 (2.7%)
Transitional Medicine (3 weeks in ED)	28 (7.7%)
Transitional Surgery (3–4 weeks in ED)	13 (3.6%)

*ED*, emergency department.

**Table 2:** Total number of patients seen per shift for emergency medicine interns: generalized estimating equation model.

Characteristic	Coefficient	Standard error	P value	95% CI
Intercept	7.16	0.15	<0.001	6.87 – 7.45
Weeks in ED	0.20	0.02	<0.001	0.17 – 0.24
Weeks of the academic year	0.01	0.01	0.130	0.00 – 0.02
Weeks in ED* Weeks of the academic year (interaction)	0.00	<0.00	0.904	0.00 – 0.01

The model estimates the per-shift productivity of an average emergency medicine (EM) intern as a function of the number of weeks spent in the emergency department (ED), the weeks of the academic year, and their interaction. For instance, an EM intern who has spent four weeks in the ED would see 7.16 + 4 * (0.20) = 7.86 patients per shift (95% CI [confidence interval] 7.55 – 8.41), without a significant difference between an intern who has just started the year (at academic week 4) or one who has had several off-service rotations (e.g., at academic week 12).

**Table 3:** Total number of patients seen per shift for off-service interns: generalized estimating equation model.

Characteristic	Coefficient	Standard error	P value	95% CI
Intercept	5.43	0.21	<0.001	5.02 – 5.84
Weeks in ED	0.46	0.11	<0.001	0.25 – 0.68
Weeks of the academic year	0.01	0.01	0.235	0.00 – 0.02
Weeks in ED* Weeks of the academic year (interaction)	0.00	<0.00	0.017	−0.02 – 0.00

The model estimates the per-shift productivity of an average off-service intern in the emergency department (ED) as a function of the number of weeks spent in the ED, the weeks of the academic year, and their interaction. For instance, an internal medicine intern who has spent two weeks in the ED would see 5.43 + 2 * (0.46) = 6.35 patients per shift (95% CI [confidence interval] 5.52 – 7.20) without a significant difference when in the academic year the rotation occurred.
